# Multi-professional meetings on health checks and communication in providing nutritional guidance for infants and toddlers in Japan: a cross-sectional, national survey-based study

**DOI:** 10.1186/s12887-018-1292-7

**Published:** 2018-10-15

**Authors:** Midori Ishikawa, Kumi Eto, Mayu Haraikawa, Kemal Sasaki, Zentaro Yamagata, Tetsuji Yokoyama, Noriko Kato, Yumiko Morinaga, Yoshihisa Yamazaki

**Affiliations:** 1 Department of Health Promotion, National Institute of Public Health, 2-3-6 Minami, Wako, Saitama, 351-0197 Japan; 2 Faculty of Nutrition, Kagawa Nutrition University, 3-9-21 Chiyoda, Sakado, Saitama, 350-0288 Japan; 3 Department of Child Studies, Faculty of Child Studies, Seitoku University, 550 Iwase, Matsudo, Chiba, 271-8555 Japan; 4 Child Health Center, Aichi Children’s Health and Medical Center, 426-7, Morioka, Obu, Aichi 474-8710 Japan; 5 Department of Food and Health Sciences, Jissen Women’s University, 4-1-1 Osakaue, Hino, Tokyo 191-8510 Japan; 6 Faculty of Medicine, University of Yamanashi, 1110 Shimokato, Chuo, Yamanashi, 409-3898 Japan; 7 Present Address: Department of Early Childhood Care and Education, Jumonji University, 2-1-28 Sugasawa, Niizashi, Saitama, 352-8510 Japan; 8 Faculty of Medicine, School of Nursing Public Health Nursing, Kagawa University, 1750-1, Ikenobe, Miki, Kita, Kagawa, 761-0793 Japan

**Keywords:** Infant health checks, Nutritional guidance, Community collaboration, Multi-professional meeting, Japan

## Abstract

**Background:**

Health personnel must provide continuous support in response to problematic results from health checks of infants and toddlers (hereinafter “infant[s]”). Among this support, it is important for health personnel to provide nutritional guidance to families as a collaborative effort between the staff from multiple disciplines and community organizations. This study aimed to clarify the factors affecting collaboration with community organizations in providing nutritional guidance to families following health checks for infants in Japan.

**Methods:**

The design of this study consisted of a cross-sectional, multilevel survey. A self-administered questionnaire was mailed to all municipalities (1741 towns and cities) in Japan to be completed by the person responsible for nutrition advice. The research was performed in August 2015. We obtained 988 valid responses (response rate of 56.7%).

To identify the factors that affect the collaboration with community organizations in providing nutritional guidance, we determined how municipalities responded to infants needing support (five items), how municipalities evaluated health guidance (five items), the number of distributed maternal and child health handbooks, and the number of infants who received follow-up evaluations.

**Results:**

The results of multivariate analyses showed that the factors related to successful community collaboration in providing nutritional guidance included holding a multi-professional staff meeting after health checks (post-conference; odds ratio [OR], 2.34; *P* = 0.001); following up children suspected of having developmental and mental disabilities or delays before entering elementary school (OR, 1.77; *P* = 0.0004); and considering dental caries data from dental checkups in providing health guidance (OR, 1.56; *P* = 0.003).

**Conclusions:**

Holding a multi-professional meeting after infant health checks (post-conference) was strongly associated with community collaboration in providing nutritional guidance for infants.

## Background

In Japan’s maternal and child health (MCH) policy, *Healthy Parents and Children 21 (Second Phase)*, the collaboration between the municipality and stakeholders in the community (e.g., kindergartens, preschools, child welfare facilities) is required to provide ongoing support for children and parents in need of health and nutritional care guidance [1]. By using a multi-professional approach—in which experts collaborate to meet the needs of the infant and parent—the appropriate quality of healthcare support can be provided [2, 3]. For example, there are cases of parents being unwilling to acknowledge concerns about the delayed development of their child based on the results of health checks. Parents may refuse to accept the guidance provided by public health nurses about their child’s development. Other issues involve children having an unbalanced diet. In such cases, dietitians are expected to provide nutritional guidance while considering the needs of the children and parents [2, 4]. Community collaboration may be required to properly respond to infants and parents needing support, health evaluation, and nutritional guidance. It is therefore important to provide nutritional guidance based on the results of infant health checks and as a collaborative effort between multi-professional staff and community organizations.

One study evaluating infant health status in the United States showed that positive weight change is related to an effective network of community collaborations [5]. Access to quality childcare services using community resources on the results of infant health status assessments is also important in improving parental behavior and reducing the need for emergency medical care for infants [6, 7]. However, no studies in Japan have investigated this topic.

In Japan, infant health checks are required by the Maternal and Child Health Law [8]. The main purpose of infant health checks and health guidance is to clarify explicit and potential health concerns and to support parents and children in dealing with these concerns [8, 9]. In 2015, the participation rate for health checks was 95.6% for infants aged 3–4 months, 95.7% for those aged 1.6 years, and 94.3% for those aged 3 years [10].

Before or after health checks in Japan, a multi-professional meeting is convened by health staff to identify health concerns (a “pre−/post-conference”). Although this form of conference is not a mandate of the designated country, this form has been adopted for information sharing among staff to connect health checks and health guidance for children in municipalities. At the pre-conference, the staff discuss pre-established concerns about each child. At the post-conference, the children who require follow-up evaluations are confirmed. In some cases, continuous support (follow-up) [11, 12], as well as the provision of nutritional guidance in conjunction with community collaborations, may be required. In such cases, it is important to share information about infant health checks with community organizations. This sharing allows deciding the best approaches to support children and parents, evaluate responses to their needs, and assess the outcomes of those activities [5]. However, few studies have investigated factors that affect the ability of health personnel and community organizations to collaborate on providing nutritional guidance to families [13]; moreover, no studies have addressed this topic in Japan.

This survey aimed to clarify the proportion of municipalities in Japan cooperating with communities in providing nutritional guidance to families whose infants received health checks, and to identify the factors related to collaboration with community organizations in providing nutritional guidance.

## Methods

### Study municipalities and procedure

This study was a cross-sectional survey of all 1741 municipalities in Japan. We used a self-administered questionnaire to determine whether infant health checks were being carried out and whether health and nutritional guidance was being provided. A copy of the questionnaire was mailed to each municipality. The department responsible for nutritional guidance following infant health checks was asked to complete the questionnaire. The director of the department then returned the questionnaire by mail or fax.

This research was performed in August 2015. In total, 1172 municipalities completed the questionnaire (response rate of 67.3%). Only questionnaires with complete responses to the items included in our analysis were considered valid, providing 988 valid questionnaires for analysis (response rate of 56.7%).

### Measurement

The questionnaire items included indicators of existing policy measures and guidelines [1, 2, 14, 15] of the Ministry of Health, Labour and Welfare; these guidelines were used in a national nutrition survey of preschool children [16]. The reliability of the items was previously confirmed [17–19].

The dependent variable was whether community collaboration is involved in providing guidance on infant nutrition. The survey respondents were asked: “Does your municipality provide nutritional guidance or education targeting infants and parents in collaboration with any organizations or groups, such as nursery schools, kindergartens, welfare facilities, or related agencies in the community, and do you evaluate those activities?” [18]. Health personnel were asked to choose an answer from the following categories: 1) We collaborate with community organizations and evaluate the activities; 2) We collaborate with community organizations but do not evaluate the activities; and 3) We do not collaborate with any organization. We designated municipalities that answered either 1 or 2 as the collaborating group and those that answered three as the non-collaborating group.

We expected certain factors to be associated with such collaboration, including methods for carrying out infant health checks, methods for determining how to respond to infants and parents needing support (five items), and methods for evaluating health guidance (five items). We also asked about potential confounding factors related to infant health checks. They included the annual number of distributed Mother and Child Health (MCH) handbooks, the annual number of infants who received a follow-up and municipality category by number of children eligible for health checks at 3 years old.

These items were chosen because they are important indicators of the health and nutrition of infants in Japan, and there are clear differences between municipalities. A copy of the MCH Handbook is provided for all pregnant women and provides a consistent health and information-provision record during pregnancy, delivery, and early years of child rearing. At every infant health check, information related to health and nutritional status are entered in the handbook, to determine suitable follow-up.

A previous study using the same data reported the validity of the municipality categories for the geographical area, the population size, and the number of children eligible for health checks at 3 years old [19]. However, the report did not show the relationship between the municipality category and community collaboration on nutritional guidance for infants. We therefore included whether category was related to the collaboration items, as a possible confounding factor.

The Appendix shows the five items addressing “how staff responded to infants and parents needing support” and “the evaluation of health guidance.” The possible answers were yes or no.

### Statistical analysis

To determine the response to infants and parents needing support (five items) and methods used to evaluate health guidance (five items), we performed an analysis comparing the answers of the collaborating and non-collaborating municipalities to the survey items about the methods used. We analyzed the results using the Cochran–Mantel–Haenszel test. Using correspondence analysis, we analyzed the relationships between the 11 items (one item on collaborating with community organizations; five items on determining responses to infants and parents; and five items on evaluating health guidance).

We performed univariate analysis for each factor using a logistic regression model based on the results of the correspondence analysis. The analyzed factors were those that were expected to be associated with collaboration in nutritional guidance: implementing a pre-conference; sharing medical records; sharing verbal information with responsible staff; implementing a post-conference; providing feedback to public health nurses and related organizations; evaluating health guidance for parents; using dental caries data in health guidance; evaluating health guidance and follow-up; and providing follow-up evaluations for infants before or after beginning elementary school.

To identify factors important for community collaboration, we performed stepwise logistic regression using the following variables: holding pre-conferences; sharing medical records; sharing verbal information with staff; holding post-conferences; providing feedback to public health nurses and related organizations; evaluating health guidance for parents; using dental caries information in health guidance; evaluating health guidance and follow-up; and providing follow-up evaluations for children who have entered or not entered elementary school. These data were adjusted for the annual number of distributed MCH handbooks, the annual number of infants who received follow-up evaluations, and the annual number of 3-year-olds who underwent health checks (model 1).

Statistical analysis was conducted using SAS software, version 9.2 (SAS Institute, Inc., Cary, NC, USA). A *P* value of < 0.05 was considered statistically significant.

## Results

Table 1 shows the comparison between community collaboration to provide nutritional guidance with distribution of MCH Handbook, number of infants who received follow-up, and municipality category by number of children eligible for health checks at 3 years old. The proportion of municipalities that collaborated with community organizations in providing nutritional guidance based on infant health checks (collaborating group) was 69.5%. The proportion that did not collaborate with any community organizations (non-collaborating group) was 30.5%. There were no significant differences between the two groups for the annual number of MCH handbooks distributed, the annual number of infants who received follow-up and the municipality category by number of children eligible for health checks at 3 years old.

**Table 1:** Comparison between community collaboration in providing nutritional guidance with distribution of MCH Handbook, number of infants who received follow-up, and municipality category by number of children eligible for health checks at 3 years old

						*n* = 988
		Collaborating group		Non-collaborating group		
		no	%	no	%	
		687	69.5	301	30.5	p
		mean	SD	mean	SD	
Number of distributions of MCH Handbook/year		693.7	1703.9	698.4	1197.5	0.240
Number of infants who received follow-up/year		58.3	140.7	70.0	164.5	0.899
Municipality category by scale of number of subjects to health checks for 3-year-old children		no	%	no	%	
*I* < 8	I	11	1.6	10	3.3	0.239
8 ≤ II < 54	II	119	17.3	43	14.3	
54 ≤ III < 391	III	301	43.8	123	40.9	
39 ≤ IV < 2916	IV	218	31.7	106	35.2	
2916 ≤ V	V	38	5.5	19	6.3	

p for homogeneity between 2 groups by Cochran-Mantel-Haenszel

Table 2 shows the relationship between community collaboration to provide nutritional guidance for infants and parents, decisions on how staff responded to infants and parents needing support (five items), and methods used to evaluate health guidance (five items).

**Table 2:** Relationship between community collaboration in nutritional guidance for infants and parents, decisions on how staff responded to infants and parents needing support, and methods used to evaluate health guidance

						*n* = 988
		Collaborating group		Non-collaborating group		
		no	%	no	%	
		687	69.5	301	30.5	p
How staff responded to infants and parents needing support (5 items)						
Implement a pre-conference	Yes	510	74.2	200	66.5	0.014
	No	177	25.8	101	33.6	
Share medical records	Yes	458	66.7	203	67.4	0.812
	No	229	33.3	98	32.6	
Share verbal information with responsible staff	Yes	426	62.0	181	60.1	0.577
	No	261	38.0	120	39.9	
Implement a post-conference	Yes	654	95.2	264	87.7	<.0001
	No	33	4.8	37	12.3	
Provide feedback to public health nurses and related organizations	Yes	547	79.6	220	73.1	0.023
	No	140	20.4	81	26.9	
Methods used to evaluate health guidance (5 items)						
Evaluate health guidance for parents	Yes	195	28.4	57	18.9	0.002
	No	492	71.6	244	81.1	
Use dental caries data in health guidance	Yes	316	46.0	97	32.2	<.0001
	No	371	54.0	204	67.8	
Provide follow-up evaluations for infants before beginning elementary school	Yes	272	69.6	74	24.6	<.0001
	No	415	60.4	227	75.4	
Provide follow-up evaluations for infants after beginning elementary school	Yes	11	1.6	4	1.3	0.747
	No	676	98.4	297	98.7	
Evaluate health guidance and follow-up	Yes	206	30.0	64	21.3	0.005
	No	481	70.0	237	78.7	

p for homogeneity between 2 groups by Cochran-Mantel-Haenszel

Collaborating municipalities were more likely to implement a pre-conference (*P* = 0.014) and a post-conference (*P* < 0.001) and to provide feedback to public health nurses (*P* = 0.023). The collaborating group also consisted of more municipalities that evaluated health guidance for parents (*P* = 0.002), used dental caries information in health guidance (*P* = 0.001), followed up children prior to elementary school (*P* = 0.001), and evaluated the relevance of health guidance and follow-ups (*P* = 0.005).

**Fig. 1:**
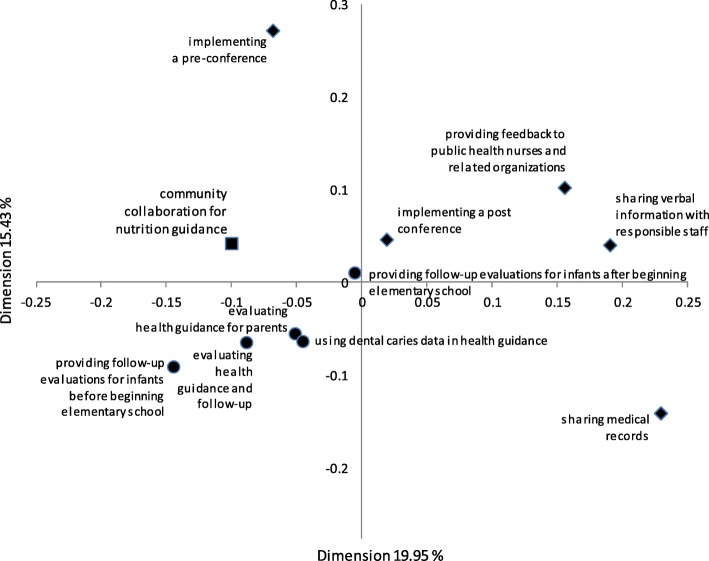
Relationships between “community collaboration for nutritional guidance”, and items on “how staff responded to infants and parents needing support” and “methods used to evaluate health guidance”, from the correspondence analysis. Key: ◆: How staff responded to infants and parents needing support (5 items); ●: Methods used to evaluate health guidance (5 items)

Figure 1 maps the results of the correspondence analysis for the relationships between community collaboration in providing nutritional guidance, decisions on the response to infants and parents needing support, and evaluation of health guidance. Items on the evaluation of health guidance were closer to community collaboration in provision of nutritional guidance than items on determining the response to infants and parents. A post-conference was closely associated with community collaboration in providing nutritional guidance in items addressing decisions on the response to infants and parents needing support. The positions of the pre-conference and post-conference were far apart, which indicates that these conferences might have different roles.

### Using logistic analysis

Table 3 shows the factors in the health check activities that were related to successful community collaboration in nutritional guidance. Following normalization (Model 1), these factors included the following: having a pre-conference (odds ratio [OR] = 1.45; 95% confidence interval [CI]: 1.08–1.94; *P* = 0.014), having a post-conference (OR = 2.82; 95% CI: 1.72–4.61; *P* < 0.001), providing feedback to public health nurses and related stakeholders (OR = 1.54; 95% CI: 1.11–2.15; *P* = 0.010), evaluating health guidance for parents needing support (OR = 1.70; 95% CI: 1.22–2.37; *P* = 0.002), using data about dental caries in health guidance (OR = 1.82; 95% CI: 1.37–2.42; *P* < 0.001), following up children before they began elementary school (OR = 2.01; 95% CI: 1.48–2.73; *P* < 0.001), and evaluating the relevance of health guidance (OR = 1.58; 95% CI: 1.14–2.18; *P* = 0.005).

**Table 3:** Factors in health check activities that are related to successful community collaboration in nutritional guidance, assessed by logistic analysis

									*n* = 988
		Model 1^a^				Stepwise			
item		OR	95% CI		p	OR	95% CI		p
How staff responded to infants and parents needing support (5 items)									
Implement a pre-conference	Yes	1.45	1.08	1.94	0.014	1.22	0.89	1.66	0.212
	No	1.00				1.00			
Share medical records	Yes	1.01	0.74	1.38	0.949				
	No	1.00							
Share verbal information with responsible staff	Yes	1.13	0.85	1.52	0.405				
	No	1.00							
Implement a post-conference	Yes	2.82	1.72	4.61	<.0001	2.34	1.39	3.94	0.001
	No	1.00				1.00			
Provide feedback to public health nurses and related organizations	Yes	1.54	1.11	2.15	0.010				
	No	1.00							
Methods used to evaluate health guidance (5 items)									
Evaluate health guidance for parents	Yes	1.70	1.22	2.37	0.002				
	No	1.00							
Use dental caries data in health guidance	Yes	1.82	1.37	2.42	<.0001	1.56	1.16	2.10	0.003
	No	1.00				1.00			
Provide follow-up evaluations for infants before beginning elementary school	Yes	2.01	1.48	2.73	<.0001	1.77	1.29	2.43	0.000
	No	1.00				1.00			
Provide follow-up evaluations for infants after beginning elementary school	Yes	1.19	0.37	3.76	0.772				
	No	1.00							
Evaluate health guidance and follow-up	Yes	1.58	1.14	2.18	0.005				
	No	1.00							

^a^Model 1: Adjusted for category of subject in 3-year-old infant health examinations, number of Mother Child Handbooks distributed per year, and number of infants followed up per year

*OR* Odds ratio, *CI* Confidence interval

In the multivariate analysis, the factors related to successful community collaboration in nutritional guidance included the following: having post-conferences (OR = 2.34; 95% CI: 1.39–3.94; *P* = 0.001), following up children before they entered elementary school (OR = 1.77; 95% CI: 1.29–2.43; *P* = 0.0004), and using data on dental caries in health guidance (OR = 1.56; 95% CI: 1.16–2.10; *P* = 0.003).

## Discussion

### Factors related to community collaboration in nutritional guidance

In this study, community collaboration in providing nutritional guidance was strongly related to the implementation of a multi-professional meetings after health checks (post-conferences). This finding is consistent with previous reports in which sharing information among staff enabled professionals to both create a shared vision, strong leadership, and broad collective goals and to clarify strengths and priorities for developing and launching projects [20]. The importance of selecting the most appropriate approach to create a collaboration has been reinforced by health data and reliable information showing the results of environmental and behavioral change [21]. If staff are unable to share child health information, then it will be unclear whether a particular response or approach is appropriate [22]. It is necessary to confirm why some municipalities do not conduct post-conferences. Post-conferences may help to establish a better system for health checks and nutritional improvement for infants.

### Multidisciplinary collaboration for nutritional support in the community

In Japan, health checks are used to make an accurate assessment of the needs of infants and their parents. Parents may continue to experience difficulties and require support throughout their child’s early years [2, 16]. It is therefore important to establish a system that enables continuous support of infants and parents in the community [2, 21, 23].

According to Japan’s national infant nutritional survey, an unbalanced diet (including snacks and soft drinks) leads to dietary issues in children. The proportion of children with such diets is significantly higher in low-income households [16]. Other studies have reported a relationship between the consumption of sugary drinks and poor eating behavior among infants and children [24–26].

One of the main objectives of community collaboration is to monitor the progress of infants and parents who require particular support in certain areas, including nutrition [21]. A multi-sectorial approach is required to address various dimensions of maternal and child healthcare, including environmental health [27].

Health professionals such as dietitians can then be involved in deciding whether further assistance is necessary or whether the issues have been resolved with the support provided [2, 5]. It is important that staff from multiple health disciplines and community groups are involved in discussions about infants and parents at post-conferences, as this involvement enables a consensus on the need for monitoring, continued support, or follow-up evaluations with home visits [6, 23].

This study found that the factors strongly related to community collaboration in providing nutritional guidance included sharing information about infant dental checkups and the results of follow-up activities before children entered elementary school. Sharing this information will lead to ongoing support throughout the child’s education [10]. The life course perspective is a critical addition to this work: it highlights the importance of ensuring that infants and children live in supportive community environments that will foster optimal health, development, and well-being throughout their lives [3, 7].

It is important to develop long-term support systems for infants and parents by sharing information, especially about food and nutrition, between community organizations such as nursery schools, pediatric clinics, kindergartens, child welfare facilities, associations for the promotion of better dietary habits, community cafeterias for children, and other governmental and non-governmental organizations [10].

This study had some limitations. First, its cross-sectional design suggests that no causal relationships can be inferred, such that reverse causation or simple correlation could account for the observations. Second, the final response rate of 56.7% was relatively low. The questionnaire was not administered to individuals, and it covered a broad range of disciplines; moreover, staff operating in different fields may have responded differently to certain items. Local government staff may also have the lacked time to complete the questionnaire during working hours and, in particular, to obtain a consensus from other staff members. Characteristics of municipalities that did not respond (around one third in total) were almost the same as those that did, including population size (small, medium and large population). One of the reasons why some municipalities did not respond may have been because that they have no full-time staff in charge of nutrition. During the study period, several municipalities responded to explain that they only had part-time staff responsible for nutritional guidance, so could not cooperate. In future surveys, it will be necessary to consider the investigation method to ensure that municipalities are not excluded because of the employment situation of staff in charge of providing nutritional guidance. Responses to the survey may also have been limited to municipalities that actively conduct health checks. A follow-up study using face-to-face interviews with health workers should be considered. Fourth, our analysis was restricted to items that could apply to all municipalities. Future investigations should examine whether the response options are unique to Japan. Municipal income was not included as a confounding factor in this survey, but should be included in a future study.

Despite these limitations, this study found relationships between community collaboration in providing nutritional guidance and the implementation of post-conferences, the follow-up evaluation of children suspected of having developmental and mental difficulties before they enter elementary school, and the use of dental checkup results in health guidance for parents and their children. The results of this study are significant because very few studies to date have examined the links between nutritional guidance and infant health checks.

## Conclusions

This study found that sharing information in a multi-professional meeting following infant health checks (a post-conference) was strongly associated with successful community collaboration in providing nutritional guidance. It highlights the importance of ensuring that infants and children live in supportive community environments that will foster optimal health, development, and well-being throughout their lives.

## Appendix

**Table 4:** Items of questionaiire on factors to be associated with community collaboration in nutritional guidance for infants and parents

How staff responded to infants and parents needing support (5 items)	
Do you share information with staff from different disciplines at meetings before health checks (pre-conference)?	
Do you share medical record information?	
Do you verbally share information with responsible public health staff?	
Do you share information with staff from different disciplines at meetings after health checks (post-conference)?	
Do you provide feedback on infant health information to the public health nurse responsible for the area of residence and related stakeholders?	
Methods used to evaluate health guidance (5 items)	
Do you evaluate the relevance of health guidance for parents who are having difficulties raising their child?	
Do you use data about dental caries from dental health checks in health guidance?	
Do you follow up children who are suspected of having developmental or mental disabilities or delays before entering elementary school?	
Do you follow up children suspected of having developmental or mental disabilities or delays after entering elementary school?	
Do you evaluate the relevance of health guidance and follow-ups, including for children who are not unhealthy but where there is some cause for concern?	

## Abbreviations

CI: Confidence interval
MCH: maternal and child health
MHLW: Ministry of Health, Labour and Welfare
OR: Odds ratio
SD: Standard deviation
